# Assessing immunization data quality from routine reports in Mozambique

**DOI:** 10.1186/1471-2458-5-108

**Published:** 2005-10-11

**Authors:** João C Mavimbe, Jørn Braa, Gunnar Bjune

**Affiliations:** 1 Faculty of Medicine, Eduardo Mondlane University, Maputo, Mozambique; 2 Faculty of Mathematics and Natural Sciences, Department of Informatics, University of Oslo, Norway; 3 Faculty of Medicine, Department of General Practice and Community Medicine, Section of International Health, University of Oslo, Norway

## Abstract

**Background:**

Worldwide immunization coverage shows an increase in the past years but the validity of the official reports for measuring change over time has been questioned. Facing this problem, donor supported initiatives like the Global Alliance for Vaccine and Immunizations, have been putting a lot of effort into assessing the quality of data used, since accurate immunization information is essential for the Expanded Program on Immunization managers to track and improve program performance. The present article, discusses the practices on record keeping, reporting and the support mechanism to ensure data quality in Mozambique.

**Methods:**

A process evaluation study was carried out in Mozambique in one district (Cuamba) in Niassa Province, between January and March 2003. The study was based on semi-structured interviews, participant observation and review of the data collection materials.

**Results:**

Differences were found for all vaccine types when comparing facility reports with the tally sheets. The same applies when comparing facility reports with district reports. The study also showed that a routine practice during supervision visits was data quality assessment for the outpatient services but none related to data consistency between the tally sheets and the facility report. For the Expanded Program on Immunization, supervisors concentrated more on the consistency checks between data in the facility reports and the number of vaccines received during the same period. Meetings were based on criticism, for example, why health workers did not reach the target. Nothing in terms of data quality was addressed nor validation rules.

**Conclusion:**

In this paper we have argued that the quality of data, and consequently of the information system, must be seen in a broader perspective not focusing only on technicalities (data collection tools and the reporting system) but also on support mechanisms. Implications of a poor data quality system will be reflected in the efficiency of health services facing increased demands, with stagnant or decreasing resources.

## Background

Immunization is an important means of controlling diseases and has been considered as the most cost effective health intervention [1]. Immunization is provided in most countries through the Expanded Program on Immunization (EPI) and as a part of the primary health care approach. Different approaches are used to enhance immunization coverage such as fixed vaccination posts, outreach services and national immunization days [2].

Worldwide immunization coverage shows an increase in the past years but the validity of the official reports for measuring change over time has been questioned [3,4]. For example, the World Health Organization (WHO) experts showed in a previous report that there was a tendency to overstate the number of fully immunized children against vaccine preventable diseases [3,5].

Facing this problem, donor supported initiatives like the Global Alliance for Vaccine and Immunizations (GAVI) have been putting a lot of effort into assessing the quality of data used, since accurate immunization information is essential for EPI managers to track and improve program performance [5].

Several studies have reported inconsistencies in data reporting as well as poor support mechanisms to ensure data quality at the district level [6-8]. For example a study done in Nepal found that data obtained from the facility registers were lower than the data reported at the district level; showing a tendency of over-reporting to the higher levels [9]. Other studies showed that errors in reporting were due to lack of supervision and feedback from the superior levels as well as inadequate incentives to health workers [6,7].

EPI in Mozambique started in 1979 following the national immunization campaign against smallpox. The EPI targets seven vaccine preventable diseases in children less than two years of age plus neonatal tetanus through immunization of pregnant women. These are Bacillus Calmette Guerin (BCG), Diphtheria, Pertussis, Tetanus and Hepatitis B (DTP+HepB), Polio and measles vaccines. The national coverage estimates for 2003 were 87% for BCG, 70% for the third dose of Polio vaccine, 72% for DTP+HepB and 77% for measles vaccine [10].

From its inception, the EPI has undergone changes along the years which led to the development of new data collection tools. The most fundamental change was full integration into primary health care services.

As with other health programs in Mozambique, EPI is hampered by chronic shortages of resources and difficult logistics due to the large geographical area, poor communications and infrastructure. This lack of resources – especially human resources – forced the Ministry of Health to adopt strategies that require inadequately trained personnel located in the most remote health facilities to cope with the challenges of general information management and routine personnel shortage [11].

With the present article, we try to answer the following research questions:

1. What are the current processes of record keeping and reporting within the EPI?

2. What are the existing support mechanisms to ensure data quality on EPI at the district level?

3. How can data quality be improved on EPI at the district level?

These questions were addressed as part of an ongoing research initiative of the Health Information System Program (HISP), which is a joint collaborative of the University of Oslo in Norway, the Eduardo Mondlane University (UEM) in Mozambique, the University of Western Cape in South Africa, and the Ministries of Health of Mozambique and South Africa. This project involved a combination of semi-structured interviews, participant observation, and review of data collection materials. The HISP program focuses on improving information to support decision making in primary health care.

## Methods

### Study scope

A process evaluation study was carried out in Mozambique in one district (Cuamba) in Niassa Province, between January and March 2003. Cuamba has an estimated population of 180,000 habitants with approximately 7200 children less than one year of age [12]. The district health infrastructure is composed of 13 health facilities of which 12 provide primary health care services, and one is a rural hospital. Cuamba at the time of the study had two medical doctors and thus most facilities were managed by paramedical staff [13].

### Selection of research site

Niassa province was chosen because the provincial directorate had identified constraining factors related to the functionality of the EPI; for example, the appearance of epidemics (like measles) in areas reporting high coverage rates [14-16]. Niassa is also among the pilot sites for HISP in Mozambique and Cuamba was selected because it constitutes a strategic point for HISP implementation as it is the referral district for the southern region of the Niassa province; Cuamba also has a relation with the Medical Faculty of the UEM, being a training site for last year medical students.

The district provides vaccination in 7 health facilities. For our study, all health facilities providing vaccination in Cuamba were selected. Since the outreach services are organized by the district headquarters and thus do not have to compete with other services and priorities like in a health facility, data from this activity has been excluded from our study.

### Sources of information

Both qualitative and quantitative approaches have been used. The qualitative materials were gathered through semi-structured interviews with open questions directed to 7 health workers from the facility level. Health workers were asked questions regarding the flow of information, the support mechanism (the quality of supervision visits, feedback and supplies) and the interaction between them and the District Health Management Team (DHMT) called NEP. Participant observation was used for the meeting held at the district level and to understand work practices at the facility level.

Quantitative materials were collected from three different sources: tally sheets, facility reports and district reports corresponding to the whole year of 2002. The assessed immunizations were BCG; the third dose of DTP+HepB; and measles vaccine in children less than one year of age.

### Information and data flows in EPI

In Mozambique, each district has an organization structure with different directorates, including the district health directorate. The district health directorate is composed of several health facilities which provide health services to the population and the DHMT that is responsible for the health management at the district level. The health facilities provide mother and child services, outpatient services and (for some facilities) the EPI services.

The official national immunization coverage figures are based on routine data produced at the facility level and by the outreach services. The outreach services are sustained by district level authorities.

**Figure 1:**
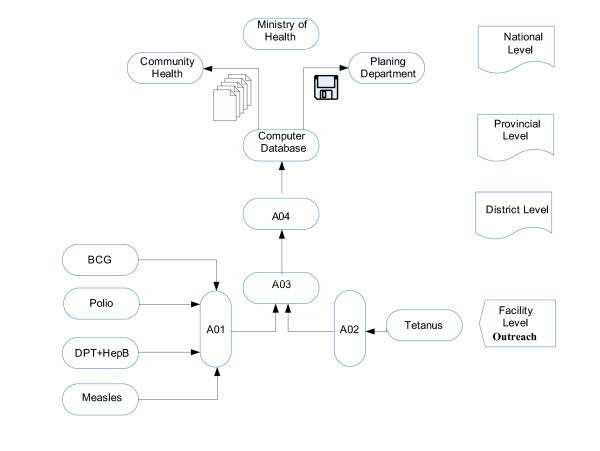
Information flow of the EPI in Mozambique.

At the first contact between a child and the health facility, the activity is recorded in the immunization tally sheet, called the A01 form for BCG, DTP+HepB (1, 2 and 3), Polio (birth, 1, 2 and 3), measles, and the A02 form for the tetanus vaccine. These constitute the basic data sources of the immunization status of the child population in the area (Figure 1).

On a monthly basis, the A01 and A02 registers are collated in a facility form (A03). The outreach services follow the same procedure as a facility, filling out a tally sheet and later the A03 form. The data are aggregated at the district level into a district form (A04) which does not discriminate information from various facilities.

The district form is sent to the provincial level on a monthly basis where the data are entered in a computer database and on a quarterly basis sent to the national level using a floppy disk (Planning Department) or in a paper format (Community Health Department).

### Data analysis

Data from the interviews were analyzed according to three different themes: information and data flows; work practices; and the existing support mechanisms. Participant observation data was used as a narrative description of the data analysis process during the quarterly meeting held at the district level. Some interviewee responses are given; these are based on verbatim notes, which are quoted in italics and have been translated from Portuguese to English.

Quantitative data (tally sheets, facility reports and district reports) were entered in a computer database using as interface Epidata ver. 3.0 (The EpiData Association) and later converted into SPSS version 11 (SPSS Inc.). All sets of data were based on the same target population from three levels: tally sheets, facility reports and district reports.

These three sets of data were analyzed by descriptive statistics. Frequencies and cross-tabulations were computed between the different sources of data. Later the data was compared and the consistency between them assessed. Significant differences were defined as those with *p < 0.05*.

Since district reports represent aggregated data, data from the outreach services were then subtracted from the district reports for comparative purposes. Due to the existing similarities in the distribution per facility, vaccine type and months, only BCG vaccine was used to illustrate the distribution per facility and per month. For the sake of anonymity the names of the health facilities were represented as A, B, C, D, E, F and G.

### Study limitations

Certain limitations of the study should be borne in mind. First of all, the findings may not be entirely applicable to other areas in Mozambique. Secondly, the sample is small and may not be entirely representative of the overall EPI program in Mozambique.

## Results

### Vaccination data at the entry point

In each facility one health worker is responsible for the EPI. It is usually the mid-wife who also holds multiple health care program responsibilities, for example, maternal health care. After vaccinating the child, the health workers take the tally sheet and register the vaccinated children.

Analyzing the data at this level, differences were found for all vaccine types when comparing facility report with the tally sheets (Table 1). Most of the facility reports showed higher values compared with the tally sheets. For example, for the BCG, the values ranged from 224% higher to 7% lower and the average was 44% higher whereas for DTP+HepB, they ranged from 474% higher to 19% lower with an average of 95% higher. For measles they ranged from 268% higher to 8% lower and the average was 72% higher for facility reports when compared with the tally sheets.

**Table 1:** Distribution of vaccines in Cuamba during 2002: differences between tally sheets and facility reports per facility

	**Number of vaccines per health facility**						
	
	A	B	C	D	E	F	G
**BCG**							
Register	89	3497	108	402	588	634	165
PHC Report	288	3537	108	448	549	655	294
Difference (%)	223.6%	1.14%	0.00%	11.44%	-6.63%	3.31%	78.18%
**DPT3**							
Register	19	2282	58	449	356	221	72
PHC Report	109	2282	62	479	288	248	206
Difference (%)	473.7%	0.0%	6.9%	6.7%	-19.1%	12.2%	186.1%
**Polio 3**							
Register	19	2294	58	449	370	230	64
PHC Report	109	2294	68	479	334	240	147
Difference (%)	473.7%	0.0%	17.2%	6.7%	-9.7%	4.3%	129.7%
**Measles**							
Register	47	1849	56	412	386	303	42
PHC Report	173	1849	60	442	356	317	135
Difference (%)	268.1%	0.0%	7.1%	7.3%	-7.8%	4.6%	221.4%

The facility analysis showed that the data increase was mainly in facilities A and facility G for all vaccine types. Conversely, facility E showed decreased values in all vaccine types. The findings showed that most of the data generated in facility B were concordant but showed an increase of 2% for BCG.

The distribution per vaccine and per month (Table 2) showed that, for facility A, a set of "zeros" was found in April and June to December. This was due to a non existent tally sheets at the facility level. A Similar situation was found in facility G for the period of April until July, September and October. For facility C, it was found that EPI services were not done from January to July due to a sick leave of the midwife. For this same facility, the study showed that there were filled tally sheets but nothing was reported to the district level during August for two vaccine types.

**Table 2:** Distribution of BCG vaccine in Cuamba during 2002: differences between tally sheets and facility reports per facility and per month (Cuamba, 2002)

	**Facility A**		**Facility B**		**Facility C**		**Facility D**		**Facility E**		**Facility F**		**Facility G**	
	**TS**	**FR**	**TS**	**FR**	**TS**	**FR**	**TS**	**FR**	**TS**	**FR**	**TS**	**FR**	**TS**	**FR**

**Jan**	18	18	251	251	0	0	35	35	32	32	47	47	0	0
**Feb**	18	18	246	246	0	0	33	33	64	64	16	16	0	0
**Mar**	19	19	282	282	0	0	41	40	29	29	15	15	0	0
**Apr**	0	19	268	308	0	0	25	25	30	30	39	39	0	28
**May**	34	34	233	233	0	0	41	41	90	90	23	23	0	50
**Jun**	0	22	246	246	0	0	30	30	38	38	36	36	0	20
**Jul**	0	51	336	336	0	0	44	44	109	86	80	80	0	20
**Aug**	0	9	344	344	12	0	27	27	51	51	33	33	86	0
**Sep**	0	29	346	346	40	52	36	47	70	70	65	86	0	51
**Oct**	0	19	340	340	31	31	0	36	55	25	118	118	0	46
**Nov**	0	34	240	240	25	25	38	38	9	9	78	78	37	37
**Dec**	0	16	365	365	0	0	52	52	11	25	84	84	42	42

TS: Tally sheets FR: Facility Reports

The data analysis also showed that there were different values in the tally sheets when compared with the facility reports characterized by increased values during September for all vaccines types in facility C as well as for facility F.

### The data at the district level

At the district level, each month, the health facilities send the facility form (A03) which is aggregated together with the outreach campaigns to make up the district average (A04). This aggregation is the responsibility of the District EPI Coordinator who, after compiling the data, sends it to the District Director to get the corresponding signature. For our study, data from the outreach campaigns are excluded.

Our study showed that numbers of all vaccines types were different in three sets of data when comparing tally sheets, regarding facility registers and district reports (Table 3). The vaccines reported by the health facilities showed an increase averaging 7% for all vaccine types compared to the tally sheets. Differences were also seen between the numbers of vaccines from the facility reports when compared with the district reports. These changes were 0.4% for BCG and 2.8% for measles, whereas the DPT+HepB showed a decrease of 0.5%.

**Table 3:** Comparison of different data sources for the EPI (Cuamba, 2002)

	**BCG**		**DPT+HepB**		**Measles**	
	
**Sources**	**Value**	**Difference (%)**	**Value**	**Difference (%)**	**Value**	**Difference (%)**
Tally Sheets (A01)	5483	-	3457	-	3095	-
Facility Reports (A03)	5879	7.2%	3674	6.3%	3332	7.7%
District Reports (A04)	5903	0.4%	3656	-0.5%	3428	2.9%

### The support mechanism

All health workers received supervision visits at intervals of less than 3 months. Supervision visits were supported by an American NGO (Medical Care Development International) which provided vehicles, fuel, and allowances. All health workers considered the supervision visits as good and as a way to increase their skills in the provision of care as well as in data analysis.

A routine practice during supervision visits was data quality assessment for the outpatient services but none related to data consistency between the tally sheets and the facility report. For the EPI, supervisors concentrated more on the consistency checks between data in the facility reports and the number of vaccines received during the same period. It was also said that supervisors were looking for miscalculations in the rows and columns of the facility report. Feedback mechanism was rare. When they existed it was based on criticism, for example "...*why aren't you reaching the target *...".

All health workers reported a lack of archiving files, punchers and staples to organize the raw data. Health workers' interaction with the district management team was characterized as "...*they want a good performance, so we provide them good data*..." therefore "...*they are not coming after us*.." but " ...*they are not providing enough supplies nor incentives to do so ...for example, to get a stapler, or a file to store the tally sheets at the facility, it is extremely difficult, ...to get fuel for the motorbike is also a very difficult process*...". It was also said that "... *if we don't provide good data showing that we are achieving the target, we are threatened not to receive the salary*....".

Errors are also related to miscalculations despite the use of a calculator. Some health workers said *"... the boss is always asking for good quality data but these calculators have very small buttons and screen, so the numbers are difficult both to type and to see*...".

Meetings took place at the district level in a monthly and quarterly basis to discuss achievements and to produce the quarterly report that has to be sent to the provincial level. Data completeness was discussed but very little about data validation. In these meetings they used a standardized spreadsheet where coverage, targets and the fulfillment index were discussed for each facility, and for each health program.

The meeting was based on criticism, for example, why health workers did not reach the target. Nothing in terms of data quality was addressed nor validation rules. For example, during one quarterly meeting where one health facility reached immunization coverage rates for BCG of 335%, for the first quarter in 2003, the health worker said that he was vaccinating the neighboring district population since they were using his services in cases of sickness. No further discussion was raised on this matter.

## Discussion

Data collection begins with the first contact between patient and health care provider. Counts of individual vaccinations are then forwarded to the next administrative level (district) where they are aggregated as a district average. Data that are recorded accurately at the facility level should correspond to data reported at the district level.

Our study showed that there were data differences between facility and district reports suggesting that there are errors in the reporting system at the facility level. These errors caused increased values for two vaccine types when comparing district reports with facility reports.

Our findings from a district perspective might not look important due to the correct data provided by facility B, which performs most of the immunizations activities in the district. On the other hand, with a facility analysis, it could be seen that facilities are over-reporting for all vaccines types and the average over-reporting ranges from 44% for BCG to 95% for DPT+HepB.

It is also important to note that analysis per facility and per month showed that the facilities reported immunized children even in the months were no children were recorded in the immunization tally sheets. This is mainly due to the way district manager's approach health workers that "did not perform well", for example, threats of salary cut.

Other abnormalities were vaccinations performed but with nothing reported to the district level. In some cases, the health worker attributed to the lack of adequate archiving materials to the absence of some tally sheets showing that the existing support mechanism is still inadequate to support the recording, storing and reporting practices.

In general, at the facility level, there is a tendency to show a "good performance" as seen by the example of the health worker that was vaccinating people at the neighboring district. This situation means that there is poor capacity to understand and to establish clear catchment areas per facility.

The notion of vaccination target and the absolute need to achieve them, since it constitutes the basis for good performance, leads to an emphasis on imperatively reaching fixed targets with success rather than effects of prevention or public health. Consequently, health workers experienced the pressure to immunize eligible children as a burden or even over reporting.

This rigidity and "discipline" imposed by the managers for target achievement may be understood as an indication of what Streefland calls, metaphorically, "a military organization model" which so often emerges in vaccination programs [17]. Further errors in the facility reporting might be added due to lack of motivation of the health personnel, lack of feedback, no concern for quality information and no cross-checking mechanism.

The system in general, as it is designed, invites "data cooking" as well as lack of interest in supporting practices such as record keeping and data use. For managers the major concern is to achieve the target; therefore, the information system is seen as an "upward system" and not as a system that may support their own work.

A common perception is that to improve accuracy and timeliness of data, redesigning the forms and data collection procedures constitutes the main solution [18]. Using this approach, implementing a register book at the facility level to ensure record keeping, could be a suggestion. However, we believe that the most important aspect is to relate information needs to interventions with focus on how information generated could be used and influence local decisions [17,18].

Some experiences, for example in Kyrgyzstan and in South Africa, showed improved data quality by giving health workers the basic skills to monitor their own work, leading to a sense of ownership of the generated information [19,20].

Different approaches can be used to improve the support mechanisms, for example, increasing the quality of the supervision visits regarding the quality of data from the tally sheet, as well as providing adequate feedback mechanism to the producers of data at the remote sites.

On the other hand, supervision visits could include a more comprehensive data analysis on EPI. It could be used as a way to do in-job training on basic concepts and monitoring indicators, a strategy used in some countries producing satisfactory results [19]. The "eyeballing" approach (a quick look at the forms), the 3C's approach (completeness, correctness and consistency) could also be promoted as a first step towards data quality improvement, and could be an essential part of health workers at the remote sites.

## Conclusion

In this paper we have argued that the quality of data, and consequently of the information system, must be seen in a broader perspective not focusing only on technicalities (data collection tools and the reporting system) but also on support mechanisms. Implications of poor data quality system will be reflected in the efficiency of health services facing increased demands, with stagnant or decreasing resources.

Within the existing study limitations, we believe that the present paper constitutes an important contribution applicable to the EPI in Mozambique and other country settings where poor data quality constitutes a bottle neck for good information systems as well as good decision making. It uses both quantitative and qualitative methods to highlight problems in a complex system where lack of resources is a constant problem.

## Competing interests

The author(s) declare that they have no competing interests.

## Authors' contributions

João Carlos de Timóteo Mavimbe participated in all phases of the study from data collection to the preparation of the manuscript and is corresponding author. Jørn Braa and Gunnar Bjune have participated on the interpretation of results and the critical revision of the paper for substantial intellectual content. All authors read and approved the final manuscript.

## Pre-publication history

The pre-publication history for this paper can be accessed here:


